# Nucleic Acid Lateral Flow Assay Implemented with Isothermal Gene Amplification of SARS-CoV-2 RNA

**DOI:** 10.3390/bios14120585

**Published:** 2024-12-01

**Authors:** Kangwuk Kyung, Hyojin Lee, Soo-Kyung Kim, Dong-Eun Kim

**Affiliations:** 1 Department of Bioscience and Biotechnology, Konkuk University, 120 Neungdong-ro, Gwangjin-gu, Seoul 05029, Republic of Korea; kkb05154@konkuk.ac.kr (K.K.); aktlapffh37@konkuk.ac.kr (H.L.); 2 Department of Laboratory Medicine, Ewha Womans University Mokdong Hospital, 1071 Anyangcheon-ro, Yangcheon-gu, Seoul 07985, Republic of Korea; skkim1@ewha.ac.kr; 3 Uniwon PharmGene Inc., 120 Neungdong-ro, Gwangjin-gu, Seoul 05029, Republic of Korea

**Keywords:** SARS-CoV-2, recombinase polymerase amplification, tailed amplicon, nucleic acid lateral flow assay

## Abstract

We developed a rapid and sensitive diagnostic platform that integrates isothermal viral gene amplification with a nucleic acid lateral flow assay (NALFA) to detect SARS-CoV-2 RNA. Isothermal gene amplification was performed by combining reverse transcription of viral RNA with recombinase polymerase amplification (RPA). In our diagnostic platform, DNA primers for the RPA reaction were modified by appending DNA tails, enabling the synthesis of tailed amplicon DNAs. These tailed amplicon DNAs were subsequently annealed to the complementary capture DNA probe affixed to the lateral flow strip during the NALFA of the reaction samples. The other side of each amplicon DNA tail was annealed to the reporter probe DNA conjugated with gold nanoparticles to visually detect the test line in the strip. This diagnostic platform reduces the time required to obtain readouts to within 1 h and can detect viral RNA concentrations as low as 3.1 cp/μL. Furthermore, when applied to nasopharyngeal clinical samples, our NALFA diagnostic platform yielded highly reliable molecular diagnostic readouts that were 100% consistent with the results of conventional RT-qPCR.

## 1. Introduction

Rapid and accurate detection of viral pathogens, such as SARS-CoV-2, is critical for effective disease management and control [1,2,3,4]. Conventional molecular diagnostics, particularly those based on quantitative polymerase chain reaction (qPCR), have been widely accepted as the gold standard [5,6,7]. However, these methods often require complex instrumentation, trained technicians, and extended processing times, which can delay diagnosis and treatment [8]. Therefore, a simple and reliable molecular diagnostic platform is needed to replace or supplement conventional qPCR tests.

Lateral flow assay-based rapid antigen tests offer a quick and straightforward method for disease diagnosis, especially in point-of-care settings [9]. These tests are generally inexpensive and provide rapid results without requiring complex procedures. However, rapid antigen tests have certain drawbacks, including lower sensitivity and a higher possibility of false negatives, particularly in cases with a low viral load in the initial stages of infection [9]. To overcome these limitations, a nucleic acid lateral flow assay (NALFA) has been developed [10,11,12,13], in which nucleic acid amplification tests (NAATs) are integrated with lateral flow assays [14,15,16]. Instead of using antibodies and haptens, the NALFA platform uses oligonucleotides with complementary binding of DNA sequences. Furthermore, this NALFA approach is more adaptable to multiplex assays than the conventional LFA based on antibody–hapten binding [17].

Isothermal gene amplification techniques simplify the molecular diagnostic process by eliminating the need for thermal cycling [18,19], which allows the application of widely distributed diagnostic methods, including point-of-care tests (POCTs). Among the isothermal gene amplification techniques, recombinase polymerase amplification (RPA) is a promising method because of the rapidity of reaction at ambient temperatures. RPA employs recombinase enzymes to achieve double-stranded DNA denaturation and strand invasion rather than relying on heat cycling, as in PCR [20]. RPA has potential benefits compared with other isothermal gene amplification methods, such as loop-mediated isothermal amplification (LAMP) [21], as it can be performed at ambient temperature (37 °C–42 °C) more rapidly and without the need for multiple complex primers. Due to its advantages over conventional LAMP, RPA was employed in this study as the NAAT for our NALFA diagnostic platform.

In our NALFA platform, amplicon DNAs with single-stranded DNA (ssDNA) tails at both ends were synthesized during the RPA reaction using primers with DNA tails (tailed forward and tailed reverse primers). The modified primers included a three-consecutive carbon (i.e., C3) spacer between the primer region and tail that served as a terminator for DNA polymerase synthesis, enabling the synthesis of amplicons with ssDNA tails [22]. During LFA, these tailed amplicons were captured by complementary DNA sequences on the strip and further annealed to a reporter probe conjugated with gold nanoparticles (AuNPs), resulting in a visual detection line. Our NALFA diagnostic platform detected SARS-CoV-2 RNA with high sensitivity and specificity, achieving results in less than 1 h and detecting viral RNA concentrations as low as 3.1 cp/μL. This platform does not require temperature transitions compared with other NAFLA methods [23,24], allowing for high-sensitivity detection of SARS-CoV-2 in a relatively short time. In addition, when applied to clinical nasopharyngeal swab samples, it yielded molecular diagnostic results that were 100% consistent with those obtained using conventional reverse transcription–quantitative real-time PCR (RT-qPCR).

## 2. Materials and Methods

### 2.1. Clinical Samples, Nucleic Acids, and Chemical Reagents

Nasopharyngeal swab samples from patients with SARS-CoV-2 infection were provided by Ewha Womans University Mokdong Hospital (Seoul, Republic of Korea) with the approval of the internal review board (IRB No. EUMC 2021-01-006). Genomic RNAs of influenza virus A (VR-1882DQTM, derived from H3N2/Wisconsin/15/2009) and influenza virus B (VR-1883DQTM, derived from Yamagata lineage/Wisconsin/1/2010) were purchased from ATCC (Manassas, VA, USA). DNA oligonucleotides for RT-RPA-LFA were purchased from IDT (Coralville, IA, USA). The detailed DNA sequences are listed in Table S1. RNase inhibitor, DNase I, and Ex Taq^®^ DNA polymerase were purchased from Takara Korea Biomedical Inc. (Seoul, Republic of Korea). M-MuLV reverse transcriptase and T7 RNA polymerase were purchased from New England Biolabs (Ipswich, MA, USA). rNTPs (A, C, G, and U) (100 mM each) were purchased from Promega (Madison, WI, USA). SSC buffer (20×) and deionized formamide were obtained from Bioneer (Daejeon, Republic of Korea). Polyvinylpyrrolidone (PVP40-100G) was purchased from Sigma-Aldrich (St. Louis, MO, USA).

### 2.2. AuNP–Reporter Probe Conjugation

The conjugation of AuNPs and reporter probes was performed using the conventional salt aging methods with slight modifications [25,26,27]. After dispensing 1.0 mL of AuNP solution (753610-25mL, Sigma-Aldrich) into a 1.5 mL tube, 1.0 µL of 10% (*w*/*v*) SDS solution was added to prevent the aggregation of AuNPs. Next, 120 µL of reporter probe (10 µM, 3′-C6 thiolated) was added, and the mixture was incubated on a MixMate^®^ shaker (Eppendorf, Germany) at 1100 rpm for 24 h. Subsequently, NaCl solution (3.5 M) was added in four increments of 70 µL each, with 20 min intervals between additions, to reach a final salt concentration of 0.7 M. The mixture was again incubated at 1100 rpm for an additional 24 h. Upon completion of the conjugation reaction, this incubation step was repeated for another 24 h. Then, the mixture was centrifuged at 21,130× *g* for 30 min at 4 °C. The supernatant was removed, and the pellet was resuspended twice in 300 µL of distilled water and finally resuspended in the same volume of conjugation pad buffer (5% sucrose, 1% BSA, and 0.5% Tween-20 in 1× PBS). After conjugation, the AuNP–reporter probe was optically characterized using a UV–vis spectrophotometer (Cary 60, Agilent, Santa Clara, CA, USA). The resuspended AuNP–reporter probe solution was then stored at 4 °C until use.

### 2.3. Fabrication of the Lateral Flow Strip

The lateral flow strip was made by manually cutting the strips to a width of 4 mm. Nitrocellulose membrane (FF120HP), Fusion 5 membrane, and CF5 dipstick pad were purchased from Whatman (Maidstone, UK). The test and control lines were drawn on the strip using an Automated Lateral Flow Reagent Dispenser (ALFRD, Claremont Biosolutions, Upland, CA, USA). Streptavidin (11 pmole) and biotinylated test/control line capture probes (22 pmole) were loaded onto the test and control lines, respectively. The membranes were then incubated for 2 h in a dry oven at 37 °C. For the sample pad, the Fusion 5 membrane was cut into strips of 1.6 cm in length. The sample pad was soaked in an appropriate amount of sample pad buffer and dried thoroughly in an oven for 2 h at 37 °C. For the conjugation pad, we used the same Fusion 5 membrane used for the sample pad, cutting it into strips of 0.9 cm in length. Subsequently, the conjugation pad was sprayed with the AuNP–reporter probe solution at a rate of 2.5 μL/mm using ALFRD and dried in a vacuum desiccator for 2 h. The prepared sample and conjugation pads were assembled onto a strip with an absorbent pad (CF5 dipstick pad). The fabricated strips were sealed and stored in the dark until further use.

### 2.4. Preparation of SARS-CoV-2 RdRp RNA

The part of the SARS-CoV-2 RNA encoding the RNA-dependent RNA polymerase (RdRp) nsP12 gene (RdRp RNA; Table S1) was synthesized in vitro using a method previously employed by our group [28]. To prepare the RdRp RNA, reverse transcription of SARS-CoV-2 RNA was performed using RT Master Mix (Toyobo, Japan). PCR amplification was conducted in a 25 μL reaction mixture containing 2 μL of cDNA, 200 nM primers (RdRp forward and reverse primers, Table S1), and 2.5 U Ex Taq^®^ DNA polymerase. The RdRp forward primer contained a 5′ overhanging T7 promoter sequence, allowing the amplified PCR product to include the T7 promoter necessary for in vitro transcription. This step was performed in a 50 μL reaction mixture containing 2.5 μL of PCR product, 2 mM rNTP mixture, 40 U RNase inhibitor, 100 U T7 RNA polymerase, and 1× T7 RNA polymerase buffer (15 mM MgCl_2_, 20 mM spermidine, 5 mM DTT, and 50 mM Tris-HCl; pH 7.5). Following incubation for 2 h at 37 °C, 5 U of DNase I was added to degrade the DNA template. Subsequently, the mixture was incubated at 37 °C for 30 min before adding 2.0 μL of EDTA (0.5 M) to inactivate the enzymes. RNA was purified via denaturing polyacrylamide gel electrophoresis (PAGE; 5%, 8 M urea). A UV–vis spectrophotometer (Ultrospec 2100 pro spectrophotometer; Biochrom Ltd., Cambridge, UK) was used to determine the concentration of the purified RNA by measuring its absorbance at 260 nm.

### 2.5. Reverse Transcription–RPA (RT-RPA)

Reverse transcription was conducted in a total volume of 10 μL, which included 2.4 μM tailed reverse primer, 100 U M-MuLV reverse transcriptase, 0.5 mM dNTP, 0.4 U/μL RNase inhibitor, and the desired RNA concentration (2.4 × 10^10^ cp/μL and 40 cp/μL in the RPA reaction mixture) in 1× M-MuLV buffer. For the amplification of RNA extracted from clinical samples, 2.0 μL of the extracted RNA was used in the reverse transcription reaction. The reaction mixture was incubated at 42 °C for 15 min, followed by enzyme inactivation by incubation at 65 °C for 20 min. The RPA reaction was performed in a separate tube following the protocol provided in the TwistAmp Basic kit (TwistDX, Cambridge, UK). The reaction mixture was prepared by first adding 29.5 μL of rehydration buffer to dissolve the lyophilized pellet. Subsequently, 480 nM tailed forward primers, 10 μL reverse transcription reaction mixture, 14 mM magnesium acetate, and distilled water were added to reach a total reaction volume of 50 μL. The mixture was then incubated at 37 °C for 15 min, followed by an enzyme inactivation step at 85 °C for 5 min.

For one-step RT-RPA, the reverse transcription and RPA reactions were conducted simultaneously in a single pot. The reactions were performed in reaction tubes using the TwistAmp Basic kit. Initially, 29.5 µL of 1× rehydration buffer was added to dissolve the lyophilized pellet. Next, the reaction mixture was supplemented with tailed forward and reverse primers at concentrations of 480 nM each, 0.8 U/µL of Recombinant RNase inhibitor, 100 U of M-MuLV reverse transcriptase, 14 mM of magnesium acetate, and RNA at the desired concentration (2.4 × 10^10^ cp/μL or 40 cp/μL). The volume of the reaction mixture was adjusted to 50 µL, and the reaction was performed at 37 °C for 15 min. Subsequently, the enzyme was quenched by heating at 85 °C for 5 min. The amplicon DNAs produced via the RPA reaction were purified using the QIAquick PCR Purification Kit (Qiagen, Hilden, Germany).

### 2.6. LFA

The RT-RPA reaction mixture obtained from either the single-pot reaction or the two-step RT-RPA was used directly in LFA without any additional purification after the reaction was completed. The samples to be analyzed in the LFA were prepared by mixing 10 µL of the RPA reaction mixture with 90 µL of running buffer (4× SSC, 1.4% (*v*/*v*) Triton X-100, 0.1% SDS, and 5% formamide). Subsequently, 100 µL of the prepared solution was applied to the strip sample pad. After a 10-minute incubation step, images of the LFA strip were captured using a smartphone camera. The test and control line signals were quantified using the ImageJ software (version 1.53).

### 2.7. Viral RNA Extraction from Clinical Samples

Viral RNA was extracted from the collected nasopharyngeal swab samples using the QIAamp Viral RNA Mini Kit (Qiagen, Hilden, Germany) according to the manufacturer’s instructions. A total of 140 µL of viral transport medium was mixed with 560 µL of carrier RNA-AVL buffer to facilitate the lysis of viral particles. The mixture was vortexed thoroughly and incubated for 10 min at room temperature to ensure complete lysis. Then, 560 µL of ethanol was added to the lysate, and the solution was transferred to a column for subsequent purification steps. After washing with the provided buffers, the extracted viral RNA was eluted in 40 µL of AVE buffer and stored at −80 °C until further analysis.

### 2.8. RT-qPCR

Clinical samples containing viral RNAs were initially subjected to reverse transcription for cDNA synthesis using ReverTra Ace™ qPCR RT Master Mix (purchased from Toyobo, Osaka, Japan). The RT-qPCR reaction mixture contained 1.0 μL of synthesized cDNA and 200 nM RT-qPCR primers (Table S1) in a 1× QuantiNova^®^ SYBR^®^ Green PCR master mix (Qiagen). The reactions were performed in a Rotor-Gene Q real-time thermocycler (Qiagen) under the following thermal cycling conditions: initial denaturation at 95 °C for 5 min; followed by 40 cycles of denaturation at 95 °C for 30 s, annealing at 60 °C for 30 s, and extension at 72 °C for 30 s; and final extension at 72 °C for 5 min. The cutoff threshold (Ct) for distinguishing between positive and negative samples was set at 31 cycles.

### 2.9. Automated Electrophoresis

The quality of the DNA samples obtained via RT-RPA reaction was assessed using a TapeStation 4150 automated electrophoresis system) equipped with ScreenTape technology (Agilent, Santa Clara, CA, USA). Individual ScreenTape lanes were filled with 1.0 µL of RT-RPA reaction mixture and 3.0 µL of buffer provided in the ScreenTape. Capillary electrophoresis was then performed for 15 min, and the processed electrophoresis image was generated using the TapeStation software (Agilent, Santa Clara, CA, USA) installed in the instrument.

### 2.10. Rapid Antigen Test Kit

Nasopharyngeal swab samples were tested for SARS-CoV-2 using two commercially available rapid antigen test (RAT) kits purchased from LUCA AICell (LUCA COVID-19 Ag Nasal Home Test, Anyang, Republic of Korea) and SD Biosensor (STANDARD Q COVID-19 Ag Test, Suwon, Republic of Korea). The samples, which were obtained from SARS-CoV-2-positive patients, were immersed in the sample extraction buffer tube provided in the kits with occasional stirring 5–10 times. Three drops of the mixed sample were then applied to the specimen loading areas of the testing devices. Ten minutes after loading, the colored band on the positive test line was visually inspected.

## 3. Results and Discussion

### 3.1. Principle of the RT-RPA-LFA Platform

**Figure 1:**
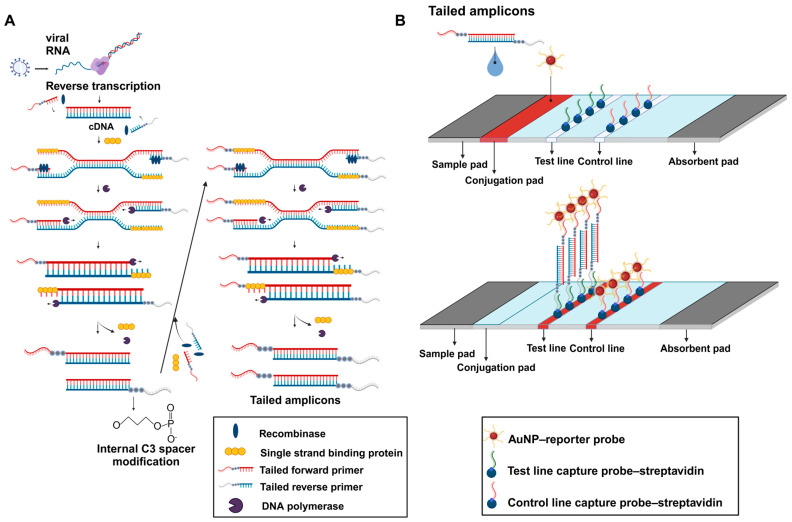
Schematic illustration of RT-RPA-LFA for SARS-CoV-2 detection. (**A**) Illustration of the RT-RPA process for synthesizing tailed amplicons. The C3 spacer, located in the middle of the tailed primers, prevents further synthesis of DNA polymerase, which allows the formation of double-tailed amplicons. (**B**) Schematics of NALFA for detecting tailed amplicons. Images were created on Biorendor.com.

RT-RPA-LFA involves the reverse transcription of viral RNA, amplification of a tailed amplicon, and visual readout, as illustrated in Figure 1. The RT-RPA reaction was performed using specially designed primers (tailed forward and reverse primers; Table S1). As the C3 spacer was inserted into the primers, the synthesized amplicon had two single-stranded DNA tails (Figure 1A). The tail attached to the forward primer was designed to bind complementarily to the reporter probe conjugated to AuNPs, whereas the tail attached to the reverse primer was designed to bind to the test line capture probe on the strip. In this platform, when the target viral RNA is amplified, the tailed amplicon connects the AuNP–reporter probe with the test line capture probe, causing the AuNPs to be collected at the test line (Figure 1B). The AuNP–reporter probe collected at the test line exhibits a red color owing to the surface plasmon resonance effect, allowing the visual detection of viral RNA. In addition, the excess AuNP–reporter probe flows past the test line and binds complementarily to the control line capture probe, giving the control line a red color.

### 3.2. Characterization of AuNP–Reporter Probe Conjugate

The AuNP–reporter probe was prepared using the salt aging method, and after conjugation, it was characterized using a UV–vis spectrophotometer (Figure S1). The highest 260 nm/520 nm values were obtained when 1.2 nmole of the reporter probe per 6.54 × 10^11^ particles was added to the binding reaction, with subsequent additions not leading to further increases. In addition, DNA amounts lower than 1.2 nmole resulted in the aggregation of AuNPs during the AuNP–reporter probe conjugation process. Therefore, a stoichiometric amount of 1.2 nmole reporter probe per 6.54 × 10^11^ gold NPs was chosen for the AuNP–DNA conjugation reaction in NALFA.

### 3.3. Validation of the NALFA Platform

**Figure 2:**
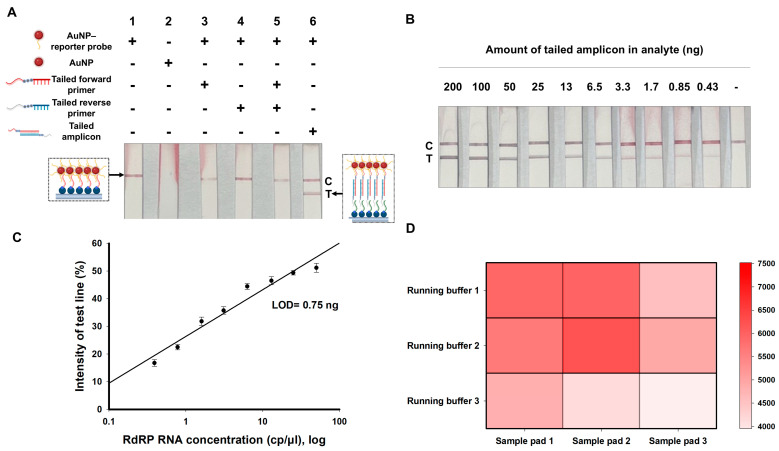
Validation of the NALFA platform. (**A**) NALFA results obtained under various experimental conditions. Each lane contained distinct samples: (1) blank sample with AuNP–reporter probe, (2) blank sample with DNA-free AuNPs, (3) tailed forward primer with AuNP–reporter probe, (4) tailed reverse primer with AuNP–reporter probe, (5) tailed forward and reverse primers with AuNP–reporter probe, and (6) purified tailed amplicons with AuNP–reporter probe. (**B**) Results of LFA with various amounts of tailed amplicons. The analytes were prepared by two-fold serial dilution of 200 ng of tailed amplicons with the rightmost strip loaded with a blank sample. Images were captured 10 min after loading the samples. (**C**) Standard curve generated from the LFA results in (**B**) based on the quantitative values of the test line signals. The limit of detection (LOD) was defined as the point at which the signal-to-background (S/B) ratio was 3, resulting in an LOD of 0.75 ng. The experiments were repeated three times (*n* = 3), and error bars display the standard deviation. The positions of the test and control lines are denoted by “T” and “C”, respectively. (**D**) A total of 200 ng of tailed amplicons was loaded onto the strip, and the intensities of test and control lines were quantified. The combined intensity values are depicted in the heat map, which showed that the highest intensities in the test and control lines occurred when using running buffer 2 and sample pad 2. Sample pad 1 was prewetted with a solution containing 0.5% BSA and 0.05% Tween-20 in 1× PBS, followed by drying in an oven at 37 °C for 2 h; sample pad 2 was treated with a solution of 0.5% PVP and 0.1% Tween-20 in 1× TBS and subjected to the same drying process to achieve optimal conditions for the assay; sample pad 3 was used directly without any prewetting or buffer application. Running buffer 1 consisted of 100 mM boric acid, 0.05% Tween-20, and 1% BSA in distilled water; running buffer 2 consisted of 4× SSC, 1.4% Triton X-100, 0.1% SDS, and 5.0% formamide in distilled water; and running buffer 3 consisted of distilled water.

The specific signals generated at the test and control lines in the NALFA, due to the tailed amplicons and the AuNP–reporter probe, were investigated (Figure 2A). As expected, a distinct red line appeared on the test line only when both tailed amplicons and AuNP–reporter probes were present. Notably, no signal was detected on the control line when DNA-free AuNPs were used in the NALFA platform. In addition, the presence of either tailed forward or tailed reverse primers did not induce any nonspecific signals.

Next, varying concentrations of tailed amplicons were applied to the NALFA platform to observe the correlation between the amount of amplicon DNA and signal intensity on the test line (Figure 2B). The signal intensity on the test line increased in proportion to the amount of tailed amplicon. The limit of detection (LOD) for tailed amplicons within the NALFA platform was 0.75 ng (Figure 2C), which was defined as the amount of tailed amplicon that produces a signal intensity three times greater than the background signal.

To ensure that our NALFA platform effectively transported tailed amplicons across the membrane and facilitated specific binding at the test and control lines, nine combinations of three different running buffers and three types of sample pads were used in the assay along with the quantification of the signal intensities of both the test and control lines (Figure 2D). Heat map analysis revealed that the combination of running buffer 2 and sample pad 2 produced the highest signal intensity. Hereafter, all NALFAs were performed under the optimized LFA condition.

### 3.4. Optimization of RT-RPA

**Figure 3:**
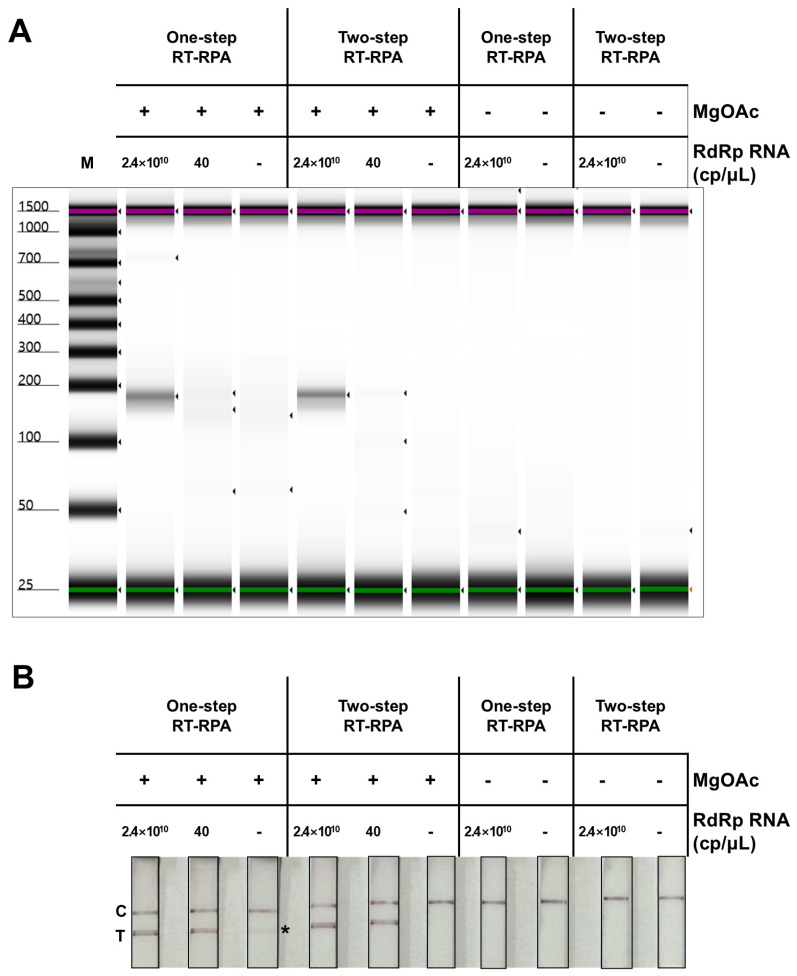
Comparison of one-step RT-RPA and two-step RT-RPA. (**A**) The products of one-step RT-RPA and two-step RT-RPA were analyzed using automated electrophoresis. For both methods, template concentrations of 2.4 × 10^10^ cp/µL, 40 cp/µL, and a no-template control were used as input RNA. In addition, a sample without MgOAc, which served as a primer for the RPA reaction, was used as a negative control. (**B**) The samples analyzed in (**A**) were further evaluated using the NALFA platform. One-step RT-RPA consistently showed detectable test line signals across all reactions including a false-positive signal (*), whereas two-step RT-RPA did not show visible test line signals in the absence of template RNA. The positions of the test and control lines are denoted by “T” and “C”, respectively.

RT-RPA, a technique that allows rapid DNA amplification at ambient temperatures, was used to synthesize tailed amplicons, which induce a red-colored signal on the test line. Reverse transcription is necessary for the amplification of viral RNA via RPA. One-step RT-RPA, in which reverse transcription and RPA occur simultaneously in a single reaction, and two-step RT-RPA, in which these processes are performed sequentially, were comparatively analyzed (Figure 3). The RPA reaction without magnesium acetate was considered a negative control. The RT-RPA experiments were conducted using two different concentrations of in vitro-transcribed RdRp RNA to evaluate the sensitivity and effectiveness of the amplification process. The first concentration was set to 2.4 × 10^10^ copies per microliter (cp/µL), which allowed clear detection of amplicon bands in electrophoresis to ensure amplification of the target RNA. The other SARS-CoV-2 RNA concentration was set to 40 cp/µL based on the RNA concentration typically detected in patient-derived SARS-CoV-2 samples via qPCR [29].

The automated electrophoresis results indicated that one-step RT-RPA generated nonspecific DNA bands in the absence of RdRp RNA (arrowheads in the fourth lane, Figure 3A). In contrast, two-step RT-RPA did not yield any detectable nonspecific DNA band in the absence of input RNA. This nonspecific amplicon was also observed as a false-positive test line band in the LFA strip of one-step RT-RPA (* in Figure 3B). Thus, the two-step RT-RPA method was selected for the isothermal gene amplification of SARS-CoV-2 RNA to detect amplicons in the NALFA platform.

### 3.5. Specificity and Limit of Detection (LOD) of RT-RPA-LFA

**Figure 4:**
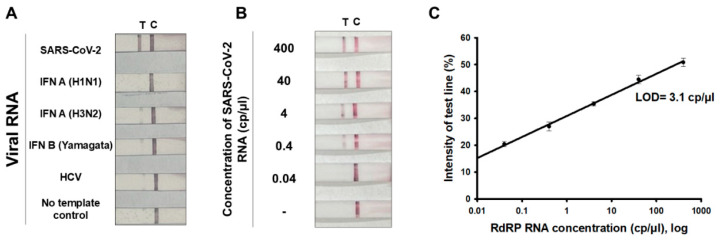
Specificity and limit of detection (LOD) of RT-RPA-LFA. (**A**) Results of the specificity test. RNAs from SARS-CoV-2, influenza virus A (H1N1 strain), influenza virus A (N3N2 strain), influenza virus B (Yamagata strain), and hepatitis C virus were applied at a concentration of 400 cp/µL for detection via RT-RPA-LFA. The test line signal was only observed in the presence of SARS-CoV-2 RNA. (**B**) Results of the LOD test. RNA was serially diluted starting from an initial concentration of 400 cp/µL, with subsequent dilutions performed in ten-fold increments down to 0.04 cp/µL. Each dilution was subjected to RT-RPA-LFA. (**C**) The standard curve displays the results from three repeated experiments (n = 3) based on (**B**), with images capturing the test line intensities expressed as a percentage. Error bars display the standard deviation. A graph was constructed, with the x-axis represented on a logarithmic scale. The LOD was defined as the point at which the signal intensity was three times as high as the background signals and was calculated as 3.1 cp/µL. The positions of the test and control lines are denoted by “T” and “C”, respectively.

After establishing the LFA platform implemented with RT-RPA (referred to as RT-RPA-LFA), the specificity and LOD of the RT-RPA-LFA platform in detecting SARS-CoV-2 were evaluated (Figure 4). In particular, the ability of the platform to selectively identify SARS-CoV-2 viral RNA among other common viruses, including influenza A (H1N1 and H3N2 strains), influenza B (Yamagata lineage), and HCV (Figure 4A), was assessed. Viral RNA samples were analyzed at a concentration of 400 cp/µL using the RT-RPA-LFA platform. Only the presence of SARS-CoV-2 RNA produced a positive signal in RT-RPA-LFA, indicating that the RT-RPA-LFA platform effectively differentiated SARS-CoV-2 from other viruses, which is essential for the accurate diagnosis of this infection.

Next, the LOD of RT-RPA-LFA was investigated by applying various doses of SARS-CoV-2 RNA copies to the platform (Figure 4B). A quantitative standard curve was constructed based on the intensity of the colored test line relative to the sum of the signal intensities of the control and test lines. The LOD was determined to be 3.1 cp/µL, corresponding to the viral RNA concentration at which the signal intensity was three times higher than the background signal (Figure 4C). This LOD was comparable to that of the RT-qPCR method, indicating the feasibility of RT-RPA-LFA as a reliable molecular diagnostic tool for SARS-CoV-2.

### 3.6. Testing of SARS-CoV-2-Positive Clinical Samples via RT-RPA-LFA

**Figure 5:**
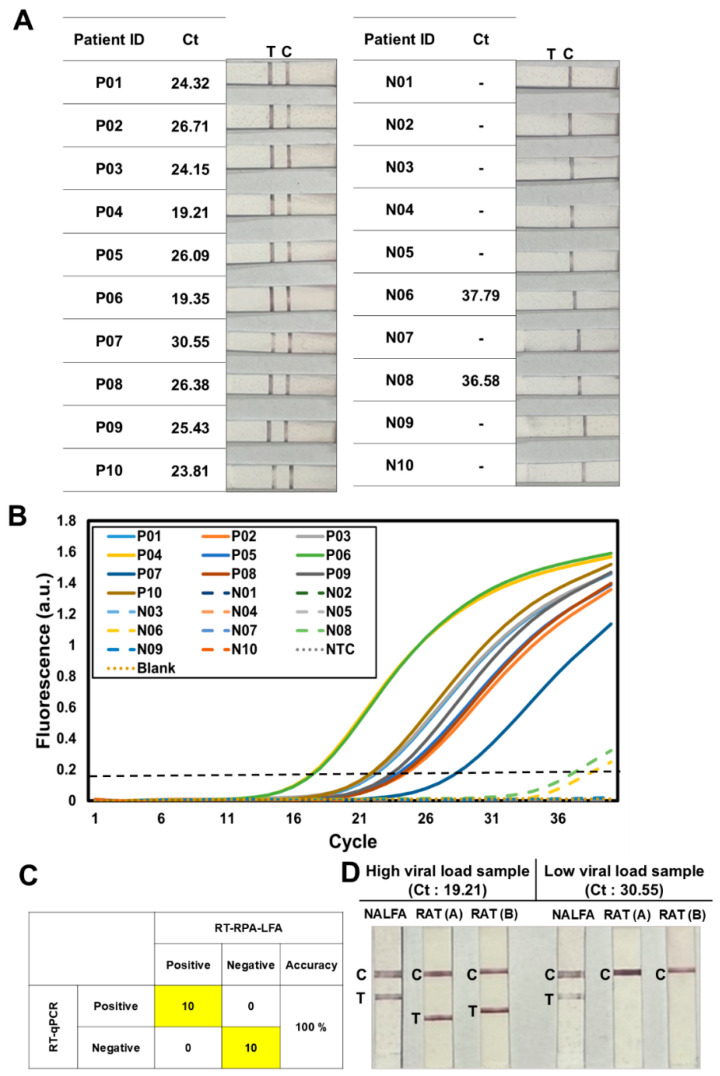
Clinical application of RT-RPA-LFA. (**A**) Results of RT-RPA-LFA performed using RNA extracted from clinical samples. In particular, there were 10 positive (P01–P10) and 10 negative nasopharyngeal swab samples (N01–N10). The RNA extracted from these patient-derived samples was also analyzed using conventional RT-qPCR, with the corresponding Ct values indicated next to each patient ID. (**B**) Graph showing the results of RT-qPCR conducted using RNA extracted from patient-derived samples. (**C**) Confusion matrix illustrating the accuracy of the RT-RPA-LFA method compared with RT-qPCR. The results obtained from the two methods achieved 100% concordance. (**D**) Comparison of the performance of RT-RPA-LFA and conventional rapid antigen tests. Clinical samples with high (Ct: 19.21) and low (Ct: 30.55) viral loads were evaluated using RT-RPA-LFA (NALFA), as well as two types of rapid antigen test kits, which are indicated by RAT(A) and RAT(B). The positions of the test and control lines are denoted by “T” and “C”, respectively.

SARS-CoV-2-positive samples were subjected to RT-RPA-LFA to evaluate the feasibility of applying this platform in clinical settings (Figure 5). Viral RNA extracted from 10 positive and 10 negative nasopharyngeal swab samples was analyzed using both RT-RPA-LFA and RT-qPCR (Figure 5A). RT-qPCR distinguished positive samples from negative ones based on a cycle threshold (Ct) value of 31 (Figure 5B). Remarkably, our RT-RPA-LFA platform provided the same diagnostic results as those obtained via RT-qPCR, demonstrating its reliability and accuracy in clinical settings (Figure 5C). In addition, the correlation between the intensity of the test line of our RT-RPA-LFA and the Ct value of RT-qPCR was analyzed, and a consistent trend was observed; the intensity of the test line increased as the Ct value decreased (Figure S2).

Next, the performance of our RT-RPA-LFA method (indicated by “NALFA” in Figure 5D) was compared with that of commercially available rapid RAT kits (purchased from two different vendors and indicated by “RAT(A)” and “RAT(B)” in Figure 5D). Two clinical samples, with high (Ct value of 19.21) and low (Ct value of 30.55) viral loads, were tested for SARS-CoV-2. The RAT kits failed to identify the virus in the sample with low viral load, whereas RT-RPA-LFA readily detected it. Thus, our RT-RPA-LFA platform demonstrated a superior capability to test clinical samples from patients in the early stages of infection compared with RATs.

Finally, the long-term stability of the developed NALFA strip was evaluated (Figure S3). When applying the diagnostic system to POCT, the long-term stability of the produced strip enhances user convenience and facilitates wider distribution. The analysis of positive and negative patient-derived samples using strips that have been aged for 1, 7, and 30 days consistently distinguished between positive and negative results, suggesting that our diagnostic platform possesses high long-term stability.

## 4. Conclusions

In this study, we developed a diagnostic platform that integrates isothermal nucleic acid amplification using RPA and an LFA to provide a rapid, sensitive, and specific method for SARS-CoV-2 detection. We achieved visual readouts of SARS-CoV-2 detection at a viral RNA concentration as low as 3.1 cp/ μL in less than 1 h. This molecular diagnostic platform demonstrated consistent performance when applied to clinical samples, with results perfectly matching those obtained via conventional RT-qPCR. Our RT-RPA-LFA method was demonstrated to be superior to RATs in detecting SARS-CoV-2 in a sample with a relatively low viral load. Thus, because of its higher sensitivity, our platform is suitable for the detection of SARS-CoV-2 in the initial stages of infection, which is not feasible via conventional RATs.

## Data Availability

Data that support the findings of this study are available within the article and Supplementary Material.

## Supplementary Materials

The following supporting information can be downloaded at https://www.mdpi.com/article/10.3390/bios14120585/s1. Table S1: Oligonucleotides (5′ to 3′) used in this study; Figure S1: Characterization of AuNP–reporter probe conjugates. (A) Images of the reaction tubes after AuNP–reporter probe conjugation. The amounts of DNA used in the AuNP–DNA conjugation reaction, with concentrations ranging from no DNA, 0.3 nmole, 0.6 nmole, 1.2 nmole, 1.8 nmole, to 2.4 nmole, as depicted in the image of the reaction tubes. (B) UV–vis spectrophotometric analysis of the products obtained from the AuNP–reporter probe conjugation reaction. The signal intensity of each sample was normalized to that of the blank sample (distilled water); Figure S2: Correlation between the intensity of the test line obtained from RT-RPA-LFA and the Ct value obtained from RT-qPCR. The results are based on the analysis results of Figure 5A; Figure S3: Long-term stability test of the NALFA strip. Positive (Ct: 19.21) and negative patient-derived samples were analyzed using strips that have been aged for 1, 7, and 30s days. The positions of the test and control lines are indicated as “T” and “C”, respectively.
