# Supplementary material for: Nucleic Acid Lateral Flow Assay Implemented with Isothermal Gene Amplification of SARS-CoV-2 RNA
Source: Biosensors (Basel). 2024 Dec 1;14(12):585. doi: 10.3390/bios14120585 (PMC11674760; doi:10.3390/bios14120585)
Supplement: Supplementary file 1 [file biosensors-14-00585-s001.zip › biosensors-3311330-supplementary.pdf]

Supplementary material

for

Nucleic acid lateral flow assay implemented with isothermal gene amplification of SARS-CoV-2 RNA

Kangwuk Kyung[1], Hyojin Lee[1], Soo-Kyung Kim[2], and Dong-Eun Kim[1,3] *

[1]Department of Bioscience and Biotechnology, Konkuk University, 120 Neungdong-ro, Gwangjin-gu, Seoul 05029, Republic of Korea. [2]Department of Laboratory Medicine, Ewha Womans University Mokdong Hospital, 1071 Anyangcheon-ro, Yang-cheon-gu, Seoul 07985, Republic of Korea. [3]Uniwon PharmGene Inc. 120 Neungdong-ro, Gwangjin-gu, Seoul 05029, Republic of Korea.

*Correspondence: kimde@konkuk.ac.kr (D.-E. Kim)

Supplementary Table and Figures

Table S1. Oligonucleotides (5′ to 3′) used in this study.

Name	Sequence (5′→3′)	Size (nt)
Tailed forward primer	TGT AAA ACG ACG GCC AGT /C3 spacer/ CTC ATC AGG AGA TGC CAC AAC TG	41
Tailed reverse primer	GTT TTC CCA GTC ACG AC /C3 spacer/ TGC GGA CAT ACT TAT CGG CAA TTT TGT TAC C	48
Test line capture probe	GTC GTG ACT GGG AAA ACT TTT TTT TTT TTT TT /Biotin-TEG/	32
Control line capture probe	TGT AAA ACG ACG GCC AGT TTT TTT TTT TTT TTT /Biotin-TEG/	33
Reporter probe	ACT GGC CGT CGT TTT ACA TTT TTT TTT TTT TTT /C6 thiol/	33
SARS-CoV-2 RdRp RNA (synthesized *in vitro*)	GGC CUC ACU UGU UCU UGC UCG CAA ACA UAC AAC GUG UUG UAG CUU GUC ACA CCG UUU CUA UAG AUU AGC UAA UGA GUG UGC UCA AGU AUU GAG UGA AAU GGU CAU GUG UGG CGG UUC ACU AUA UGU UAA ACC AGG UGG AAC CUC AUC AGG AGA UGC CAC AAC UGC UUA UGC UAA UAG UGU UUU UAA CAU UUG UCA AGC UGU CAC GGC CAA UGU UAA UGC ACU UUU AUC UAC UGA UGG UAA CAA AAU UGC CGA UAA GUA UGU CCG CAA UUU ACA ACA CAG ACU UUA	285
RdRp forward primer (+ T7 promotor, underlined)	TAA TAC GAC TCA CTA TAG GCC TCA CTT GTT CTT GCT C	37
RdRp reverse primer	TAA AGT CTG TGT TGT AAA TTG CGG AC	26
RT-qPCR forward primer	GCT CGC AAA CAT ACA ACG TG	20
RT-qPCR reverse primer	CAT TAA CAT TGG CCG TGA CA	20

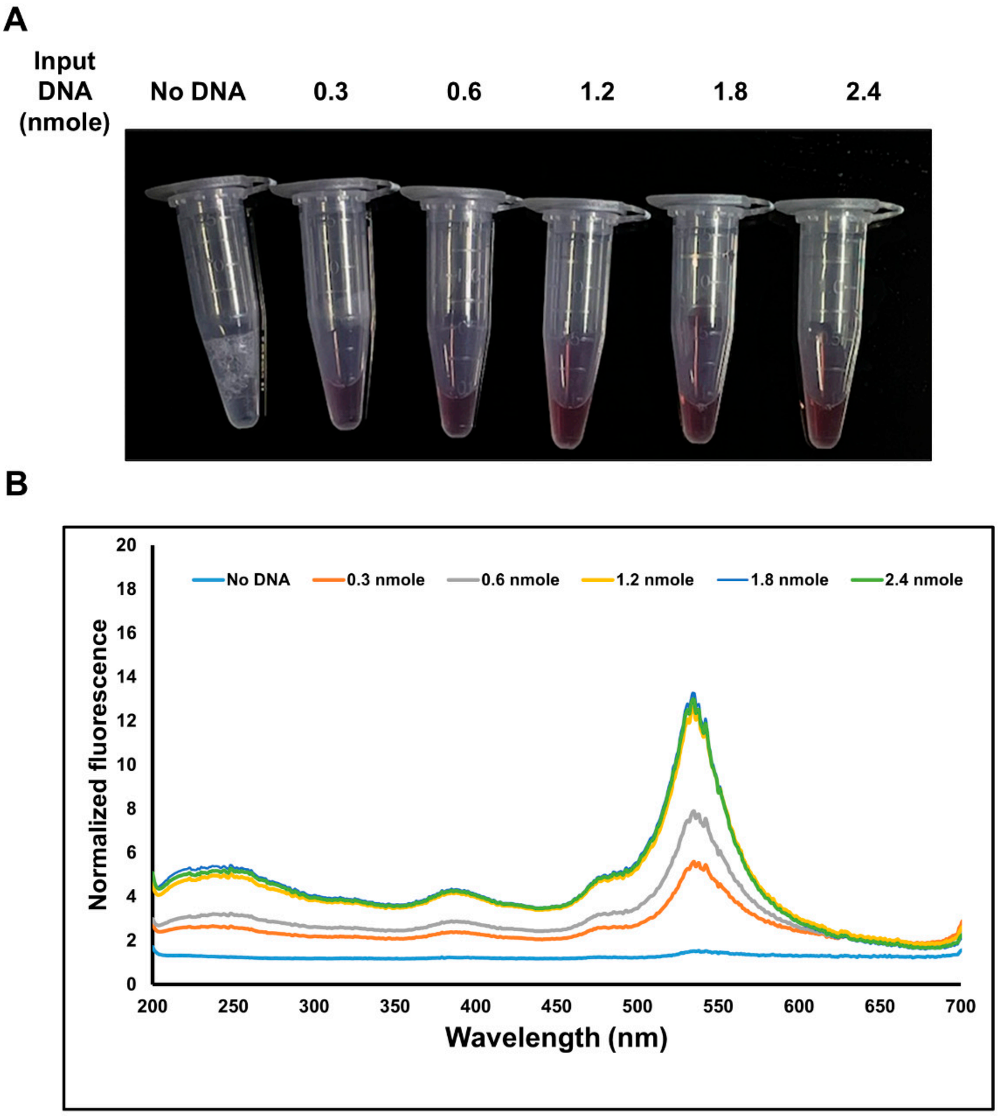

Figure S1. Characterization of AuNP–reporter probe conjugates. **(A)** Images of the reaction tubes after AuNP–reporter probe conjugation. The amounts of DNA used in the AuNP–DNA conjugation reaction, at concentrations of 0, 0.3, 0.6, 1.2, 1.8 and 2.4 nmole, are shown in the image of the reaction tubes. **(B)** UV–vis spectrophotometric analysis of the products obtained from the AuNP–reporter probe conjugation reaction. The signal intensity of each sample was normalized to that of the blank sample (distilled water).

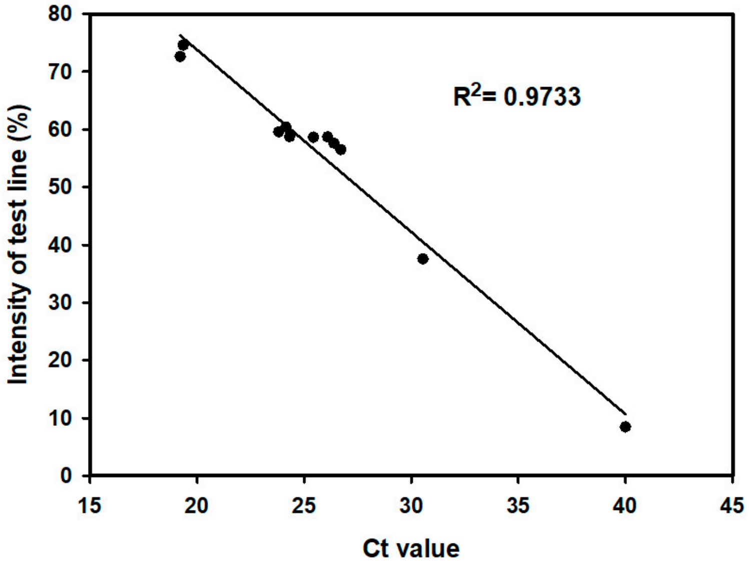

Figure S2. Correlation between the intensity of the test line obtained from RT-RPA-LFA and the Ct value obtained from RT-qPCR. The results are based on the analysis results of Figure 5A.

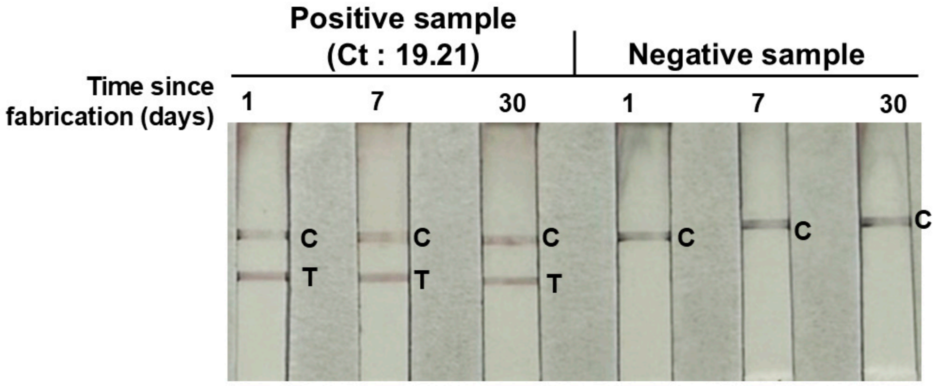

Figure S3. Long-term stability test of the NALFA strip. Positive (Ct: 19.21) and negative patient-derived samples were analyzed using strips that have been 1, 7, and 30 days. The positions of the test and control lines are indicated as "T" and "C," respectively.
